# Emerging applications of fluorescence spectroscopy in medical microbiology field

**DOI:** 10.1186/1479-5876-7-99

**Published:** 2009-11-26

**Authors:** Aamir Shahzad, Gottfried Köhler, Martin Knapp, Erwin Gaubitzer, Martin Puchinger, Michael Edetsberger

**Affiliations:** 1 Max F. Perutz Laboratories, Department of Structural Biology and Biomolecular Chemistry, University of Vienna, Vienna, Austria; 2 OnkoTec GmbH. Waidhofen/Thaya, Vienna, Austria

## Abstract

There are many diagnostic techniques and methods available for diagnosis of medically important microorganisms like bacteria, viruses, fungi and parasites. But, almost all these techniques and methods have some limitations or inconvenience. Most of these techniques are laborious, time consuming and with chances of false positive or false negative results. It warrants the need of a diagnostic technique which can overcome these limitations and problems. At present, there is emerging trend to use Fluorescence spectroscopy as a diagnostic as well as research tool in many fields of medical sciences. Here, we will critically discuss research studies which propose that Fluorescence spectroscopy may be an excellent diagnostic as well as excellent research tool in medical microbiology field with high sensitivity and specificity.

## Discussion

### Limitations/Drawback of current diagnostic Tools

Infectious diseases are caused by microorganisms such as bacteria, viruses, fungi and parasites. Infectious diseases are major killer around the world especially in developing countries. Infectious diseases were responsible for 14.7 million deaths around the world in 2002 [1] major portion of health care budget are allocated for diagnosis and treatment of infectious diseases. There are many diagnostic methods and techniques available for microorganisms associated diseases. These include morphological examination by microscopy, culture examination, biochemical tests, and histopathology approach. There are modern sophisticated methods are also available like PCR, ELISA, molecular DNA analysis. But, there are many limitations and drawbacks associated with these diagnostic techniques. These techniques are time consuming, laborious and require many reagents [2]. Also, some techniques lack high sensitivity and specificity which warrants the need for a new diagnostic technique with high sensitivity and specificity.

Current traditional diagnostic techniques and methods for diagnosis of microorganisms like bacteria take normally at least one day. Also, Antibiotic sensitivity testing is also required by physicians to choose specific antibiotic for treating infection. This sensitivity testing usually takes one more day. Bacteria are cultured for at least one day and then diagnosis is made. This causes delay in start of specific treatment. As a result physicians usually prescribe broad spectrum antibiotics which are unnecessary and very expensive for patients. Also, microorganisms have unique mechanisms to develop resistance for antimicrobial treatment. It justify for fast diagnosis of microorganisms and start of specific treatment as soon as possible.

### Fluorescence spectroscopy

Fluorescence spectroscopy seems to be promising diagnostic technique with fast and rapid diagnosis ability. Studies indicate high sensitivity and specificity rate which makes Fluorescence spectroscopy an ideal diagnostic tool for medical microbiology field. But, there is need for further studies and clinical trials to validate this new diagnostic technique.

At present, Fluorescence spectroscopy is being applied in medical microbiology field for various purposes. There are many studies which indicate that Fluorescence spectroscopy is promising diagnostic technique with high sensitivity and specificity for microorganisms associated diseases diagnosis with the help of spectroscopic fingerprints. Also, Fluorescence spectroscopy and Fluorescence correlation spectroscopy (FCS) may be applied to understand various pathophysiological steps of various microorganisms [3,4].

**Figure 1:**
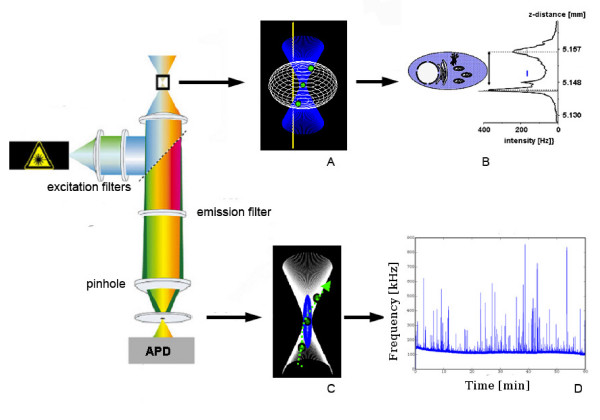
**FCS instrumentation for use in living cells**. On the left hand, there is schematic FCS setup including laser excitation filters, emission filters, confocal pin hole and single photon detector (APD). To use this setup, the laser beam is positioned inside the cell (A). The exact position of the focus is established by performing a Z scan (B). The pin hole cuts out a defined focal element from the laser focus (C). The Fluorescence signals from fluorescenct entities moving through the focal element are recorded by the single photon detector, resulting in a Fluorescence trace (D). (Source: Shahzad A, Edetsberger M, Köhler G. Fluorescence Spectroscopy: An emerging excellent diagnostic tool in Medical Sciences. Applied Spectroscopy Reviews J (In press).

Fluorescence spectroscopy is a type of electromagnetic spectroscopy which analyzes fluorescence from a sample. The sample is excited by using a beam of light which results in emission of light of a lower energy resulting in an emission spectrum which is used to interpret results [5]. Fluorescence correlation spectroscopy (FCS), a technique basically used for spatial and temporal analysis of molecular interactions of extremely low concentrated biomolecules in solution. (Figure 1) FCS measures both the average number of molecules in the detection volume and the diffusion time of the molecules through the open detection volume [6]. As the diffusion speed is directly correlated with the molecular mass and shape of the fluorescent molecule, it is possible to study the complex formation between a small fluorescent labeled and a big unlabelled molecule [7].

### Fluorescence correlation spectroscopy (FCS)

**Figure 2:**
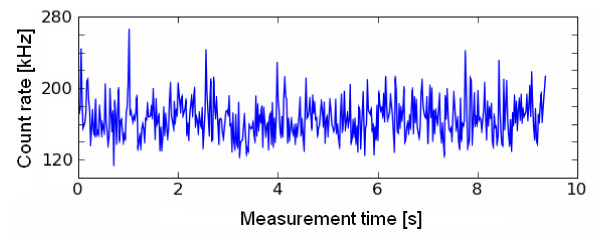
**Fluorescence fluctuations measured by FCS**. (Source: Shahzad A, Edetsberger M, Köhler G. Fluorescence Spectroscopy: An emerging excellent diagnostic tool in Medical Sciences. Applied Spectroscopy Reviews J (In press).

**Figure 3:**
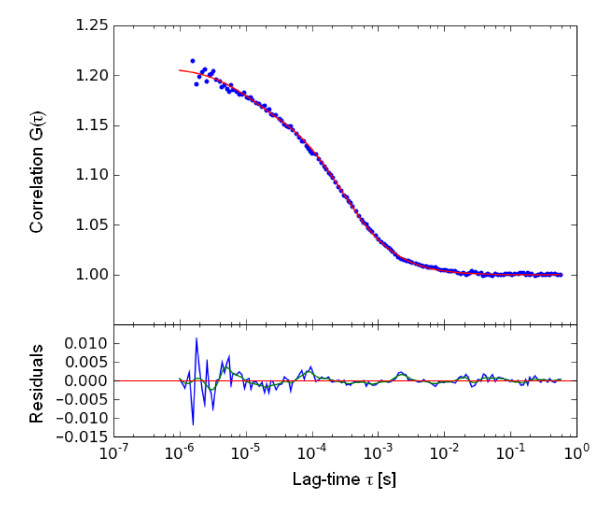
**Autocorrelation function generated from fluorescence fluctuations (Fig. 2) This function is used to determine the average diffusion time of the particles inside the FCS focus during measurement time**. (Source: Shahzad A, Edetsberger M, Köhler G. Fluorescence Spectroscopy: An emerging excellent diagnostic tool in Medical Sciences. Applied Spectroscopy Reviews J (In press).

Fluorescence correlation spectroscopy (FCS) use the basic principle that a fluorescing molecule shows a specific free diffusion velocity which is directly correlated with its size. So, bigger the molecule, slower it will diffuse through a given spherical volume. This basic phenomenon of molecules is used in FCS to study protein-protein interactions, attachment and many more. (Figure 1) Fluorescence Correlation Spectroscopy (FCS) uses statistical deviations of the fluctuations in fluorescence in order to study dynamic molecular events, such as diffusion or conformational fluctuations of bio molecules or artificial particles. (Figure 2) Mainly, the auto correlation function (ACF) is used to extract the number and diffusion coefficient of fluorescent particles diffusing through the focus volume. (Figure 3) These all properties of FCS make it an excellent diagnostic and research tool for many medically important diseases. Various properties of FCS make it an ideal tool for understanding various pathophysiological processes involved with microbial infectious diseases. An excellent advantage of FCS is that it requires very low concentrations and amounts of samples, as compared to routinely used techniques which require high concentration of diagnostic sample.

Tryptophan which is fluorophore in UV is present in both viruses and host bacterial protein. Indole group of tryptophan residues are major source of UV absorbance and emission in proteins. Tryptophan in pure water emits at 353 nm [8]. Tryptophan emission is strongly associated with its local environment. Many phenomena such as protein-protein association result in spectral shifts in tryptophan emission [8]. It is proposed that emission and excitation spectral differences may be due to presence of different environments of tryptophan residues in specific proteins of microorganism's cells [9].

### Diagnostic Applications

At present, many studies reported successful application of Fluorescence spectroscopy as a diagnostic tool for different bacteria at genus, species and group level by use of spectral fingerprints [10-12]. Spectral studies for black pigmented bacilli which are a group of oral bacteria showed significant difference in spectral signatures of each bacterium [12]. Fluorescent profiles of Bacteria which are responsible for otitis Media in children: S. *pneumoniae, S. aureus, M. catarrhalis*, and *H. influenzae *have been studied. These studies proved that each bacterium produce a different specific Fluorescence profile. The data indicate that it may be an excellent non invasive fluorescence based diagnostic technique for otitis media [12]. In another study; three different bacterial species (*Escherichia coli*, EC, *Enterococcus faecalis*, EF and *Staphylococcus aureus*, SA) were rapidly identified by autofluorescence spectrum differences coupled with Principal Components Analysis (PCA) technique. These studies proposed that bacteria can be rapidly diagnosed with sensitivity and specificity higher then 90% [13].

### Bacterial taxonomy

Fluorescence spectroscopy was utilized for pseudomonad taxonomic purpose at species and genus level [14]. Results proved that Fluorescence spectroscopy may be an excellent tool in polyphasic approach to pseudomonad taxonomy. This approach provide more information as compared to rRNA and DNA bacterial homology grouping as they provide more information about strain relatedness and good differentiation between strains which are difficult to differentiate on PCR and API 20NE identification methods [14].

### Fungal applications

Fungal infections are common in many diseases like diabetes, many types of cancers, endocrinopathies, and patients on prolonged antibiotics or immunosuppressive drugs. Diagnosis of fungal infection is made either by morphological examination of fungi or by biochemical and molecular biology techniques [15]. These techniques may not differentiate between different types of yeast. There are studies which have utilized spectroscopic fingerprints method for rapid diagnosis of different fungi such as yeast, *Microsporum gypseum*, *Microsporum canis*, *Trichophyton schoenleinii*, *Trichophyton rubrum*, *Epidermophyton floccosum *and *Fusarium solani *[9,16].

### Viral Applications

Studies indicate that Fluorescence spectroscopy may be a novel diagnostic tool to detect viruses. Also viral infections of cells can be monitored by Fluorescence spectroscopy [3]. These studies were carried out on viruses from cystovirus family and pseudomonad host cells. Tryptophan which is fluorophore in UV is present in both viruses and host bacterial protein. Within proteins, tryptophan structural environment is not same and this structural difference is responsible for specific spectroscopic signatures [3]. This property can be used to monitor viral attachment process and to study the release of progeny virus particles by analysis of tryptophan emission spectra during infection process.

In author's Lab, Fluorescence correlation spectroscopy (FCS) has been applied successfully to understand human rhino virus-receptor interaction [17]. These experiments provide informative data for understanding virus-receptor interactions. Fluorescence correlation spectroscopy (FCS) studies revealed different binding modes for an icosahedral virus along the five-fold symmetry axis. We proposed that Fluorescence correlation spectroscopy (FCS) may be a valuable technique to study various receptor binding affinities of viruses.

#### Future Research

Spectroscopic technique may be automatized which can then process many diagnostic samples at the same time. Also, fiber optic systems may be integrated with this spectroscopic technique to diagnose microorganisms in vivo. By this modification, infections in many body parts can be detected with ease. Further research is required to establish flexible and portable spectroscopic devices which can be integrated in daily medical practice.

There is need for reference libraries for spectral signatures of individual microorganism. This will be very helpful for comparison with spectral signatures from an unknown microorganism sample. But, there are many questions which remain to be answered like if biological sample contains more than one microorganism, then how it will affect the spectral signature appearance and how to interpret these spectral for making definite diagnosis. Also, microorganisms like bacteria have many chemicals which are same like in human cells and in extracellular space, thus body fluids samples may contain same chemicals as found in microorganisms. As a result, it may interfere with spectroscopic spectral analysis and may be a hurdle to reach on definite diagnosis. This justifies the need for studies which can enable to make distinction between microorganism and human cells. Also, future studies should be directed to determine the specific spectral regions which will be suitable for identification of specific microorganisms. It will help to design invasive and non invasive techniques for microorganism's diagnosis inside the body cavities by use of fiber optic devices.

## Conclusion

At present, nearly all the diagnostic techniques and methods used for microorganism's diagnosis are not perfect and have some limitations. There is great need for a diagnostic technique which can overcome limitations and drawbacks of commonly used microbiological techniques and methods. Studies indicate that Fluorescence spectroscopy have great potential to become an excellent and perfect diagnostic technique for microorganisms. In many research studies, fluorescence emission spectra derived from autofluorescence property of many medically important bacteria make it possible to distinguish between various bacterial species and also enable to classify the bacteria into genus, species and groups. Recent research studies indicate that virus particles can be monitored inside cells and various processes of viral infections can be detected by means of Fluorescence spectroscopy. Difference between fungal microorganisms like yeast can be made easily by use of spectroscopic fingerprinting. Future clinical trials on large scale should be performed to validate Fluorescence spectroscopy as a diagnostic tool for microorganisms. Flexible and portable spectroscopic devices should be design which can be integrated in routine medical practice.

Overall, emerging research studies and data points that Fluorescence spectroscopy is a potential diagnostic tool for microorganisms. Based on these data and research studies, we expect that in near future, Fluorescence spectroscopy will be available as a routine diagnostic tool for microorganisms in daily medical practice. Ultimately, Patients will benefit from its low cost, fast processing and high sensitivity properties. In the long term, spectroscopy fingerprinting may become an excellent tool to classify microorganisms into their respective groups, genus and species level. This will be very promising system with high sensitivity and high specificity for microorganisms classification.

## Competing interests

The authors declare that they have no competing interests.

## Authors' contributions

All authors participated in the preparation of the manuscript, and read and approved the final manuscript.
